# Eco-friendly fabrication of copper oxide nanoparticles using peel extract of *Citrus aurantium* for the efficient degradation of methylene blue dye

**DOI:** 10.1038/s41598-024-79589-4

**Published:** 2024-11-25

**Authors:** Alaa I. Khedr, Mohamed H. H. Ali

**Affiliations:** National Institute of Oceanography and Fisheries, Cairo, Egypt

**Keywords:** CuO, Photocatalysis, *Citrus aurantium*, Green synthesis, Methylene blue, Chemistry, Nanoscience and technology

## Abstract

This study presents a simple, sustainable, eco-friendly approach for synthesizing copper oxide (CuO) nanoparticles using *Citrus aurantium* peel extract as a natural reducing and stabilizing agent. The synthesized CuO and CuO-OP were characterized using various techniques, including surface area measurement (S_BET_), X-ray diffraction (XRD), Fourier-transform infrared spectroscopy (FTIR), diffuse reflectance spectroscopy (DRS), scanning electron microscopy (SEM) coupled with energy dispersive X-ray spectroscopy (EDX), and high resolution transmission electron microscope (HRTEM). DRS analysis determines band gap energy (E_g_) of 1.7 eV for CuO and 1.6 eV for CuO-OP. FTIR confirmed the presence of Cu–O bond groups. The XRD and HRTEM results revealed monoclinic and spherical nanostructures, with average particle sizes ranging from 53.25 to 68.02 nm, as determined via Scherer’s equation. EDX analysis indicated incorporation of carbon (1.6%) and nitrogen (0.3%) from the peel extract. The synthesized CuO and CuO-OP NPs exhibited excellent photocatalytic efficiencies for methylene blue dye under UV irradiation, reaching 95.34 and 97.5%, respectively, under optimal conditions; the initial dye concentration was 100 mg/L, the pH was 10, the catalyst dosage was 1 g/L, and the contact time was 120 min. Isothermal studies showed that the adsorption of MB onto the nanoparticles followed the Freundlich isotherm model (R^2^ = 0.97 and 0.96). Kinetic studies indicated that the degradation followed pseudo-first-order kinetics, with rate constants (K_1_) of 0.0255 min^−1^ for CuO and 0.033 min^−1^ for CuO-OP. The sorption capacities were calculated as 98.19 mg/g for CuO and 123.1 mg/g for CuO-OP. The energy values obtained from the Dubinin–Radushkevich isotherm were 707.11 and 912.87 KJ mol^−1^, suggesting that chemisorption was the dominant mechanism.

## Introduction

Environmental pollution poses a threat to the health of all living organisms, including people. The spread of different pollutants into the ecosystem can lead to severe consequences, especially contamination of drinking water and ecosystem degradation. Dyes are major contributors to water pollution, particularly in places where industries such as paint, pigment, and textile production are concentrated^1,2^. In recent decades, various methods have been used globally to remove dyes, including adsorption, reverse osmosis, nanofiltration membrane, oxidation, microbiological degradation, flocculation, ion exchange, Fenton reactions, and photocatalytic degradation^3–7^.

Photodegradation is the most practical method that achieves a balance between cost and efficiency while having little and insignificant environmental impact^8,9^. A wide range of metal oxides, such as TiO_2_, ZnO, SnO_2_, Fe_2_O_3_, CoO, NiO, and CuO have been extensively utilized to eliminate harmful dyes from wastewater^10–12^. Therefore, developing effective and sustainable photocatalytic technologies has become an important priority in environmental research to address the hazards posed by pollutants.

The green synthesis of nanoparticles is a sustainable and eco-friendly approach that has garnered significant attention because of its great advantages. Unlike traditional chemical and physical methods, which often involve the release of toxic chemicals, high energy consumption and hazardous by products, green synthesis relies on natural resources such as plant extracts, microorganisms, or biodegradable materials as reducing and stabilizing agents^1^. This technique provides more advantages than chemical methods, as it enables the controlled preparation of nanoparticles with specific sizes and shapes while also being feasible and cost-effective^13^ and can be easily scaled up for large-scale synthesis at relatively low pressure and temperature conditions^14^. Green biosynthesis nanotechnology focuses on the synthesis of nanomaterials using bio organism extracts, including microbial enzymes or plant phytochemicals, to initiate biological processes. Moreover, green-synthesized NPs often display improved biocompatibility and reduced toxicity, making them ideal for biomedical applications^15,16^. The use of abundant, renewable natural materials further enhances the cost-effectiveness of this approach, presenting a cleaner and more ethical path for developing nanotechnology innovations.

Purkait et al.^17^ utilized *Trema orientalis* leaf extract as a reducing and capping agent for the green synthesis of ZnO NPs, resulting in a crystallite size of 24 nm. Varadavenkatesan et al.^16^ used an ecofriendly approach for the green synthesis of silver nanoparticles using *Thunbergia grandiflora* leaf extract for the photocatalytic degradation of Acid Red 88 and Methylene Blue. Their findings showed that the degradation process followed the first-order kinetics with kinetic constants of 0.18 min^-1^ and 0.14 min^-1^, respectively. Similarly, Selvara et al.^18^ synthesized stable silver nanoparticles using *Tabebuia aurea* leaf extract with a spherical morphology and a mean size of 48.68 nm, demonstrating excellent reduction of the Congo Red and Acid Blue 113 dyes with NaBH_4_, with first-order kinetic constants of 0.2723 and 0.1335 min^−1^ respectively. Furthermore, Purkait et al.^19^ synthesized green TiO_2_ NPs with particle sizes ranging from 88 to 94 nm using *Trema Orientalis*(L) leaf extract for the photodegradation of Zoxamide [3, 5-dichloro-N-(3-chloro-1-ethyl-1-methyl-2-oxopropyl)-4 methyl benzamide].

Copper oxide nanoparticles have attracted significant interest in the field of metal oxide nanoparticles because of their affordability, ease of synthesis, low bandgap (1.7 eV), excellent photocatalytic properties, and nontoxic characteristics^20^. CuO is an effective semiconductor used in catalytic photodegradation processes to break down organic contaminants. Furthermore, it possesses antibacterial, antifungal, antimicrobial, biocidal, superconductive, gas sensing, and optical capabilities^21^. CuO NPs can be synthesized using several methods such as the sonochemical method, sol‒gel, electrochemical, microwave irradiation, alkoxide-based, and solid‒state reaction techniques^22^. Similarly, these nanoparticles are also produced through green techniques involving plants, fungi, algae, and other natural product extracts^23^.

Oranges (Citrus fruit) are a valuable source of vitamin C and several essential phytonutrients^24^. Orange peel (OP) typically contains numerous bioactive compounds and phytoconstituents with strong reducing properties. These include phenols, terpenoids, flavonoids and polysaccharides, which facilitate the reduction of metal ions by denoting electrons, these compounds accumulate on the surface of nanoparticles, acting as capping agents that prevent agglomeration, thereby enhancing their stability over time^24^. Green extracts also improve biocompatibility and enhance performance in several applications like drug delivery, environmental remediation, and catalysis. Additionally, these components can enhance the production of nanoparticles, eliminate free radicals, and improve photodegradation. Several studies have described the use of orange peel extract (OP) for the synthesis of cobalt^25^, platinum^26^, and zinc oxide nanoparticles^27^.

Singh et al.^28^ produced copper oxide nanoparticles (CuO NPs) by using the leaf extract of *Psidium guajava* as reducing, and capping agent. These nanoparticles have shown an exceptional ability to degrade reactive yellow-160 (81%) and Nile blue (93%) through photocatalysis within 120 min. Khani et al.^29^ employed an extract *of Ziziphus spina christi* (L.) to synthesize copper NPs. These nanoparticles exhibited exceptional adsorption properties for crystal violet, with a high adsorption efficiency of 37.5 mg g^−1^ within 7.5 min. Sukumar et al. produced CuO NPs using an extract of *Caesalpinia bonducella* seed^30^. Sangeetha & Abarna^9^ utilized the lemon peel extract to synthesize CuO NPs for photocatalytic degradation of methyl orange.

The main objectives of this study are as follows: (1) To explore the utilization of orange peel extract (OP) in environmentally friendly biosynthesis; (2) to characterize and compare the structural composition of chemically synthesized CuO and CuO-OP fabricated via green methods; (3) to demonstrate the photocatalytic efficiency of methylene blue dye using both CuO and CuO-OP; and (4) to optimize the experimental conditions of photocatalysis, such as pH, exposure time, initial dye concentration, and catalyst amount.

## Results and discussion

### Characterization

#### N_2_ adsorption-desorption isotherm

**Fig. 1:**
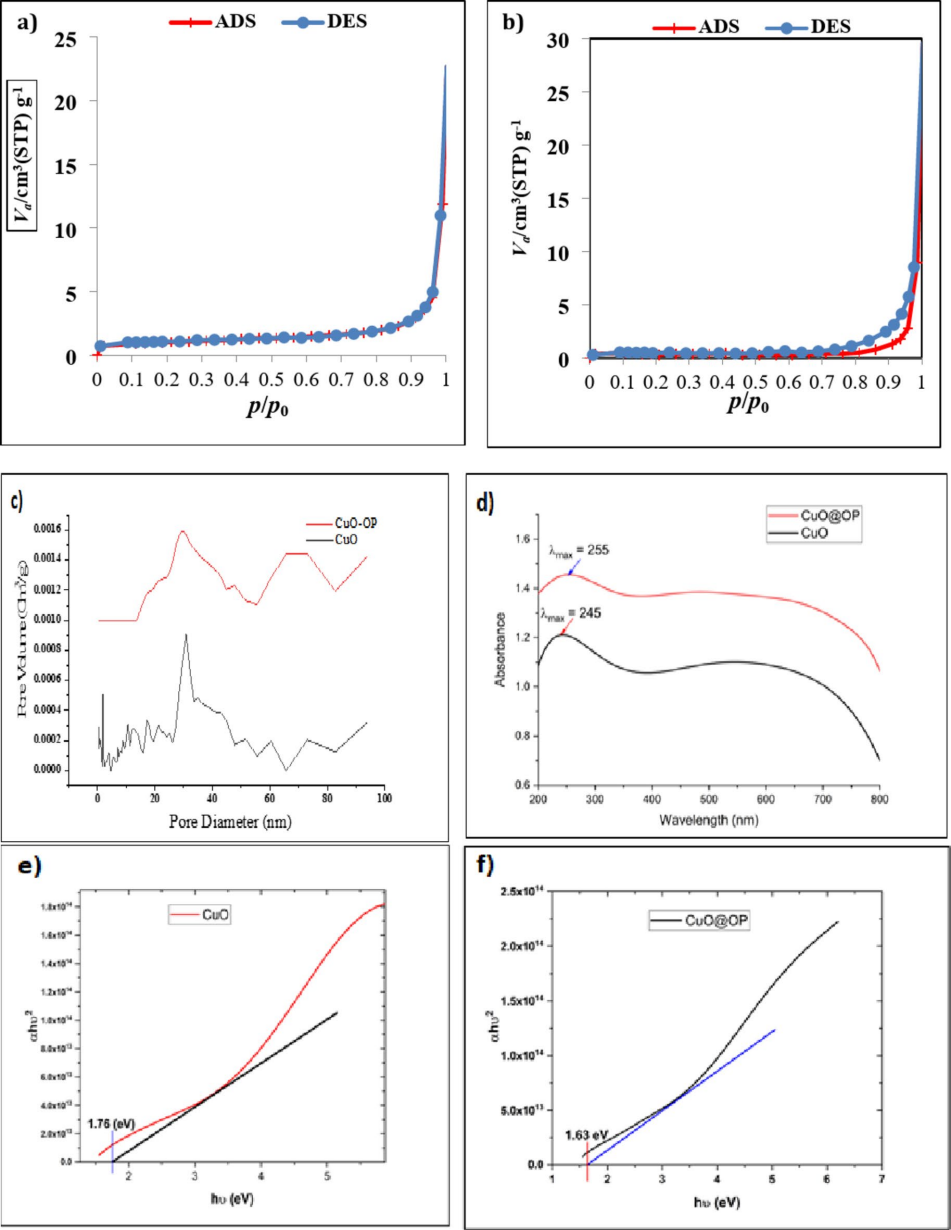
(**a**) Nitrogen adsorption/desorption isotherm of CuO, (**b**) Nitrogen adsorption/desorption isotherm of CuO-OP, (**c**) pore size distributions of CuO and CuO-OP, (**d**) UV‒Vis DRS plot of CuO and CuO-OP, (**e**) Tauc’s plot of (α***hυ***)^2^ vs. ***hυ*** of CuO NPs and (**f**) Tauc’s plot of CuO-OP NPs.

Surface area estimation is an important factor in the photocatalysis process. The N_2_ adsorption-desorption isotherms exhibit a type IV categorization with an H3 hysteresis loop for CuO and CuO-OP NPs on the basis of IUPAC classification (Fig. 1a,b). This categorization is observed within a range of P/P_0_ values from 0.85 to 0.99 and is indicative of a mesoporous structure^31,32^. The increased surface area allows for enhance the availability of binding sites where dye molecules can be adsorbed and active radicals can be generated. Furthermore, the extended surface area resulted in accelerated diffusion and mass transfer of reactive species, leading to enhanced photocatalytic reaction kinetics^33,34^. The surface area of CuO NPs was 3.48 m^2^g^−1^, with an average pore volume and diameter of 0.019 cm^3^g^−1^ and 21.87 nm, respectively. After orange peel extract was added, the surface area decreased to 1.82 m^2^g^−1^, with an average pore volume and diameter of 0.020 cm^3^g^−1^ and 44.64 nm, respectively (Table 1). Despite, the surface area decreased upon addition of orange peel extract, the catalytic activity increased. This may be attributed to the increasing number of multifunctional groups that increase the average volume and diameter of the surface pore^35^. Baylan et al.^36^ synthesized CuO NPs with a surface area of 1.7 m^2^g^−1^. Fig. 1c shows the pore size distributions of the CuO and CuO-OP NPs. CuO NPs showed various modes centered at 2, 10.6, 12.79, 17.52, 21.3 and 31.15 nm which are referred to as mesoporous surfaces. Other modes centered at 51.48, 60.51, 72.90 and 93.94 nm are referred to as macroporous structures. Upon the addition of the OP extract, a homogeneous pore size distribution was noticed. The pore size distributed between mesopores (2–50 nm) was cenetred at 29.68 and 47.71 nm, with the existence of macropores (> 50 nm) at 65.77, 72.73 and 93.82 nm (Fig. 1c).

**Table 1:** Specific surface area, radius and pore volume of CuO and CuO-OP.

	V_m_ (cm^3^(STP)g^−1^)	S_BET_ (m^2^ g^−1^)	Total pore volume (cm^3^ g^−1^)	Average pore diameter (nm)
CuO	0.8	3.48	0.019	21.88
CuO-OP	0.42	1.82	0.02	44.64

#### Diffuse reflectance spectroscopy (DRS)

UV–Vis diffuse reflectance spectroscopy (DRS) is a useful tool to examine the optical properties of photocatalysts and determining their bandgap energies. Distinct absorptions are observed at 245 and 255 nm for CuO and CuO-OP, respectively (Fig. 1d). The band gap energy (E_g_) was calculated via the Tauc plot and Eq. (1), where *h* is Planck’s constant, α is the absorption coefficient, ${\upupsilon}$ is the frequency and λ is the absorption wavelength in nm.

1 $${(\propto {h}{\upupsilon })}^{2}={A}({h}{\upupsilon }-{{E}}_{{g}})$$

2 $${\upupsilon }= \frac{1}{{\lambda }}$$

The E_g_ was estimated to be 1.7 eV for CuO (Fig. 1e). Upon addition of the OP extract to the CuO structure, the band gap was reduced to 1.6 eV (Fig. 1f). The low E_g_ enhances the ability of green NPs to degrade high organic pollutant contents in a reasonable amount of time. Alsalama et al.^37^ estimated the band gap energy of CuO to be 1.6, 1.7 and 1.8 eV at different calcination temperatures of 350, 450 and 550 °C, respectively. On the other hand, Kir et al.^38^ used lemon peel extract to synthesize CuO/PEG and CuO NPs, which exhibited a spherical morphology, average sizes of 45 and 34 nm, and optical bandgap energies of 1.5 and 1.2 eV, respectively.

#### X-ray diffraction (XRD)

**Fig. 2:**
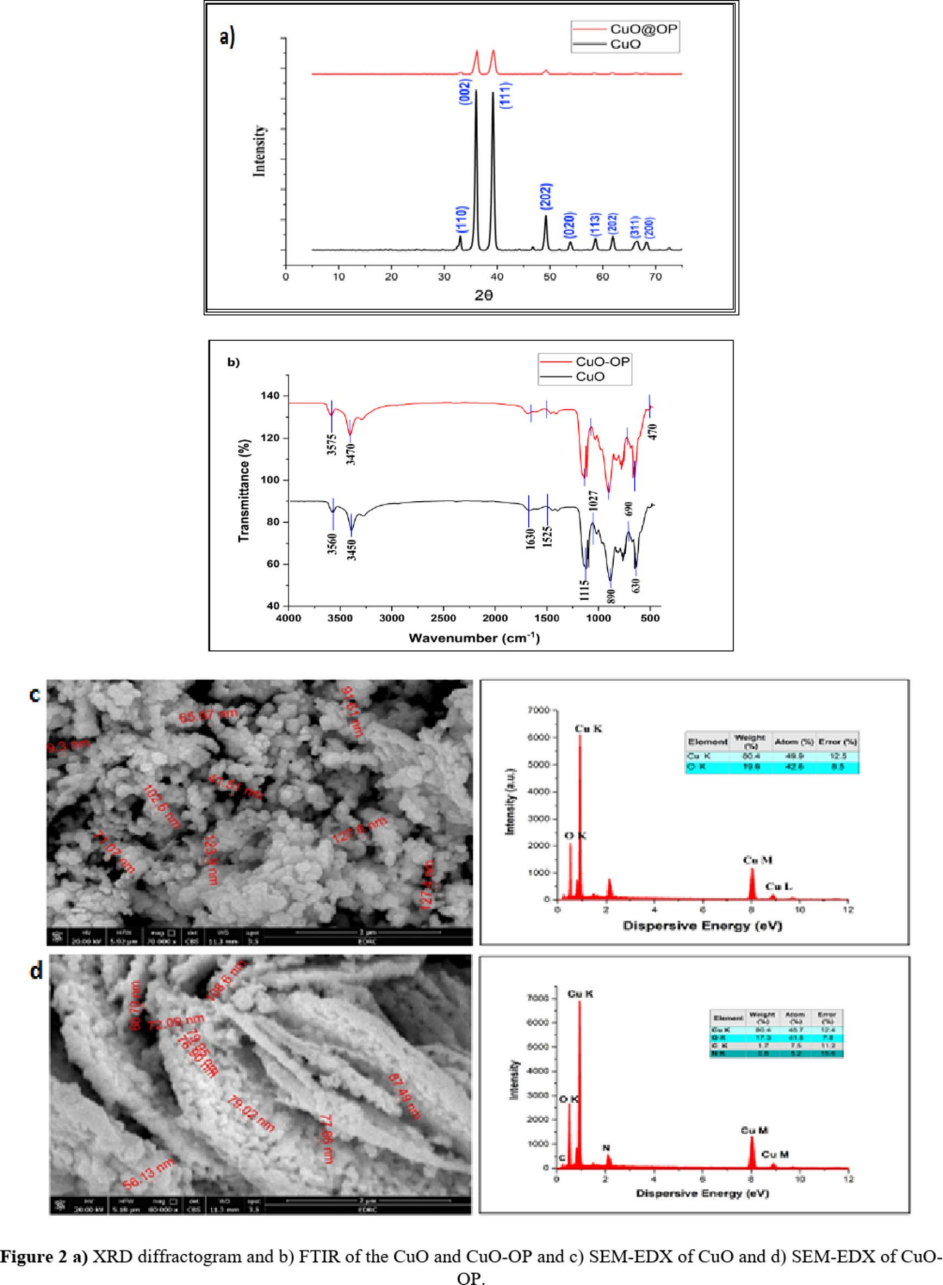
(**a**) XRD diffractogram and (**b**) FTIR of the CuO and CuO-OP and (**c**) SEM-EDX of CuO and (**d**) SEM-EDX of CuO-OP.

XRD is a common technique used to analyse nanomaterials and determine their crystallite properties, such as their crystal structure, average size, phases, and lattice parameters^32^. Several peaks at 32.99, 35.99, 39.19, 49.20, 53.85, 58.59, 61.89, 66.33, and 68.28° were assigned to hkl values of (110), (002), (111), (202), (020), (202), (113), (311) and (200), respectively, revealing successful synthesis of the CuO NPs. The crystal structure of CuO and CuO-OP NPs is typically monoclinic, and the size of CuO crystals was calculated via the Scherer equation (Eq. 3), yielding values ranging from 53.25 to 68.02 nm with an average of 61.23 nm. The measured d-spacing values were approximately 3.4846 Å and 3.2357 Å, with lattice constants (a) of 5.25 and 5.15 Å, respectively, reflecting variations in crystallinity and particle size during synthesis^39^. These findings closely align with those of Chowdhury et al.^40^ who reported d-spacing and lattice constant values of 0.2 nm and 0.34 nm for green-synthesized CuO nanoparticles using *Lantana camara*. Additionally, a slight shift in 2θ with reduced intensity was observed upon the addition of the OP extract (Fig. 2a). The primary mode of crystal growth was mainly observed along the (002) and (111) planes for both CuO and CuO-OP, revealing the existence of a cubic spherical structure in the fabricated oxides.

#### FTIR

FT-IR analysis was conducted to characterize the surface properties of the synthesized CuO nanoparticles (Fig. 2b). The FT-IR spectrum of pure CuO NPs shows two peaks at 3560 and 3450 cm^−1^ which are assigned to the bending vibration OH group^41^, there are remarkable bands appeared at 1630, and 1525 cm^−1^ which are attributed to stretched carbonyl group (C=O) coupled with COO– bond of amide I, another obvious peak appeared at 1115 cm^−1^ which assigned to bending NH coupled with stretching CN of amide II^42^. The peaks appeared at 1115 and 1027 cm^−1^ assigned for C–O stretching of alcoholic compounds^43^. The strong peaks appeared at 890, 690, 630 and 470 cm^−1^ related to the vibrations of the Cu–O indicating the successfully formation of CuO NPs^44^. For biosynthetic CuO-OP NPs, there are relatively small shifts in the absorption CuO-OP bands were observed as a consequence of the presence of flavonoid and phenolic groups attached to the surface of CuO NPs after the biosynthesis process using the OP extract (Fig. 2b).

#### SEM-EDX

SEM analysis revealed the external morphology of the prepared nano-oxides^45^. Fig. 2c shows the presence of various pores with numerous separated islands of nanoparticle accumulation at the CuO surface. These pores play essential roles in the adsorption and photodegradation of dye molecules on the CuO surface. Furthermore, various channels with different cavities are observed in the SEM image of CuO-OP (Fig. 2d). These created interconnected channels expedite the diffusion of pollutant particles within the dye solution. This enhanced accessibility to the nanoparticle surfaces, accelerated adsorption and subsequently improved photodegradation. Furthermore, this spongy porous nature of the catalysts increases the overall surface area, providing more sites for pollutant molecules to interact with and degrade^46^. A greater surface area provides more contact points for the pollutant molecules, facilitating their adsorption and photodegradation onto the nanoparticles. Furthermore, the EDX spectrograms revealed the presence of distinctive peaks of Cu (89.5%) and O (9.5%), with no foreign peaks, confirming the excellent purity of the sample (Fig. 2c). On the other hand, impregnating the OP extract led to the presence of some minor peaks of C (1.6%), and N (0.3%) in addition to those of Cu (81.1%), and O (17.1%). The presence of C and N improved the surface morphology of the catalyst for dye photodegradation (Fig. 2d).

#### HRTEM-SEAD

**Fig. 3:**
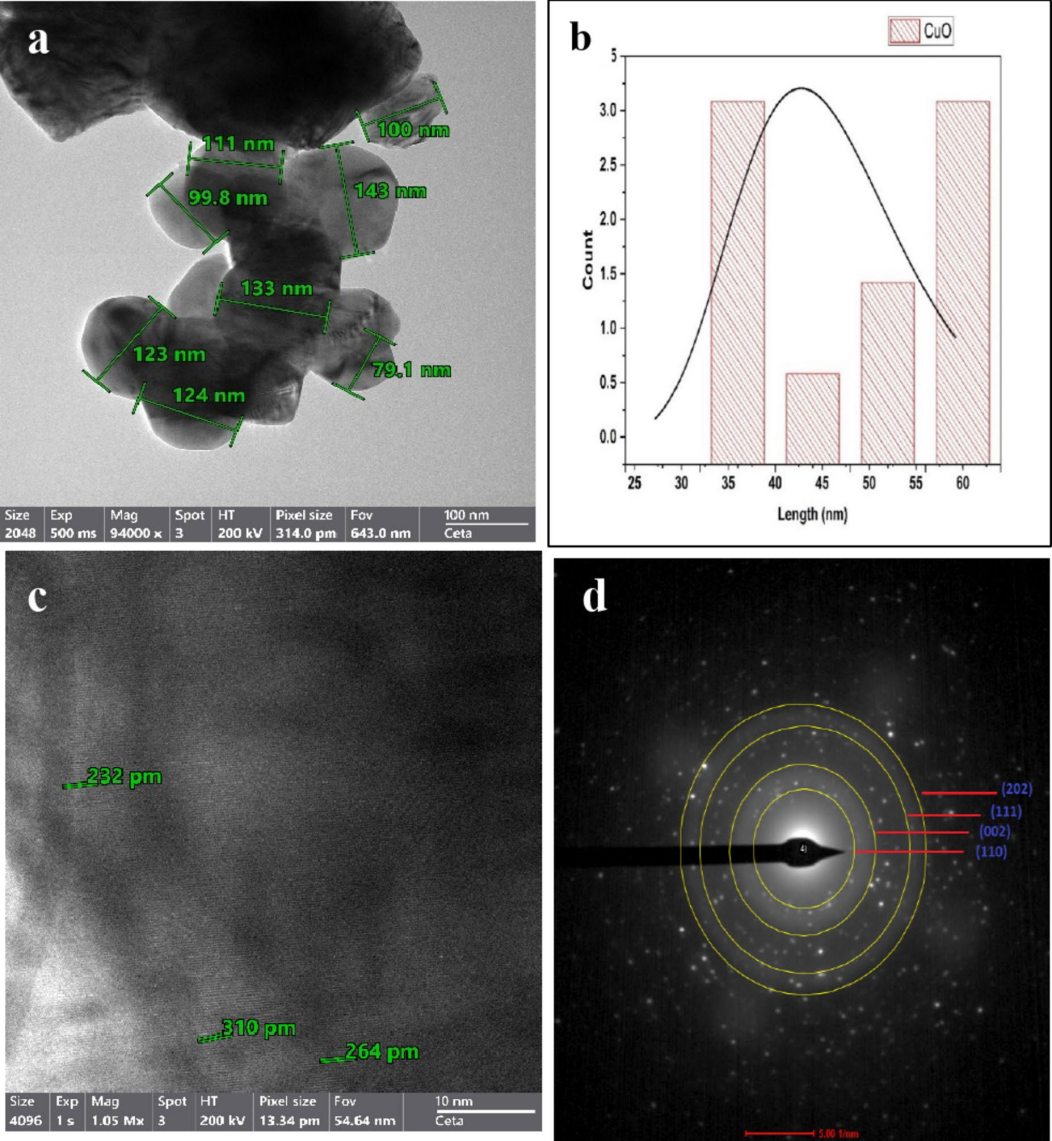
TEM images of CuO indicating (**a**) HRTEM, (**b**) particle size distribution, (**c**) d-spacing and (**d**) SAED.

**Fig. 4:**
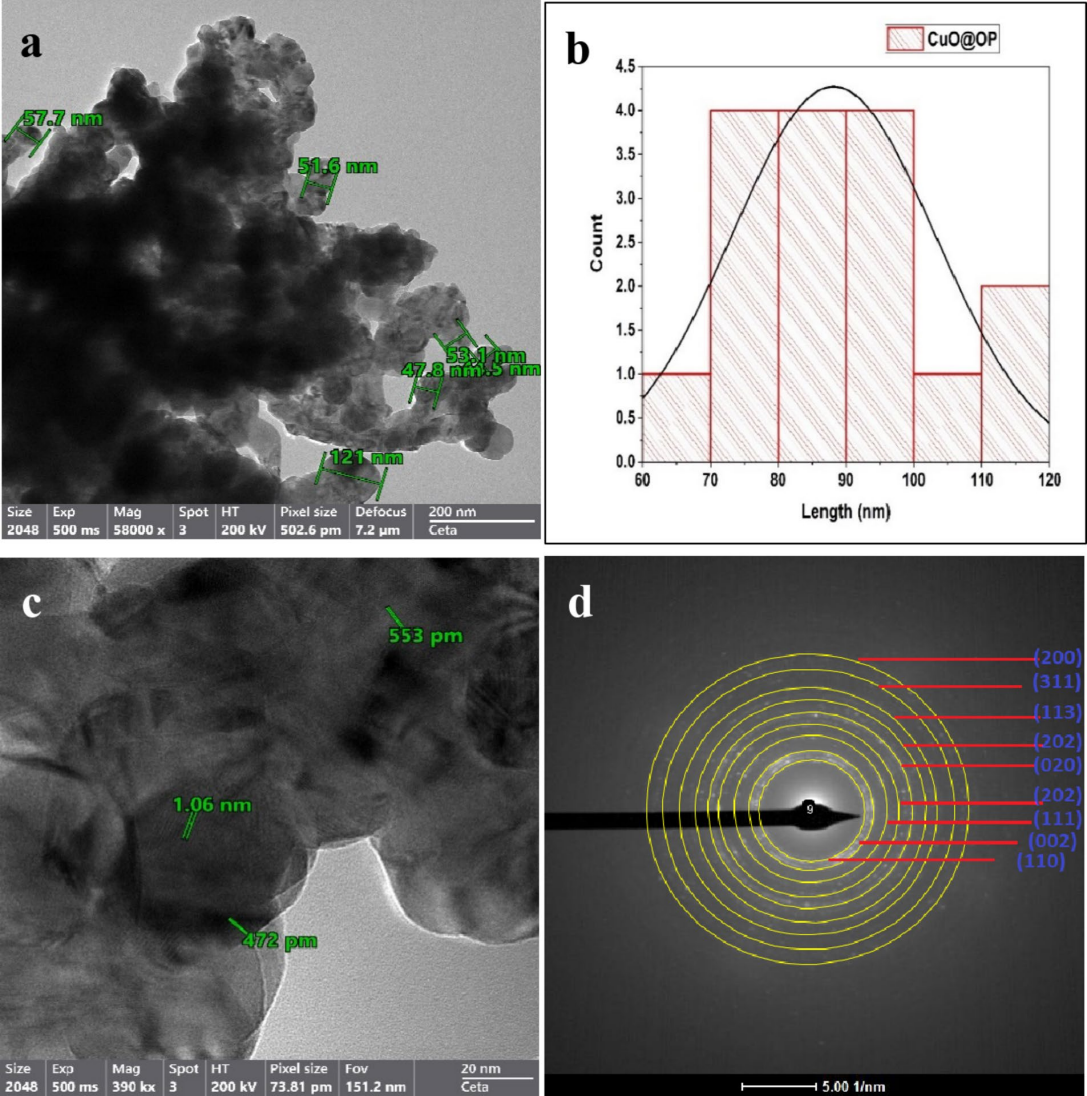
TEM images of CuO-OP NPs indicating (**a**) HRTEM, (**b**) particle size distribution, (**c**) d-spacing and (**d**) SAED.

TEM images provide additional insight into the structure of the synthesized photocatalysts and depict the distribution of the particle size. The TEM micrographs demonstrate the spherical cubic morphology of both CuO and CuO-OP (Figs. 3 and 4). The nanoparticles are arranged into clusters, forming numerous pores and cavities that enhance the adsorption and photodegradation of dye molecules (Figs. 3 and 4). The particle size varied within 79.1 -143 and 47.8–121 nm for CuO and CuO-OP, respectively. It is evident that OP extract aids in stabilizing the nanometal oxide particles during preparation, resulting in a decreased particle size. To obtain a more detailed view of the atomic structure, HRTEM imaging was performed, revealing interplanar distances of 0.232–0.310 and 0.472–1.06 nm, for CuO and CuO-OP NPs respectively. This finding provides more evidence for the crystal structure of the nanomaterials (Figs. 3 and 4). Furthermore, selected area electron diffraction (SEAD) revealed circular rings, revealing the polycrystalline nature of both CuO and CuO-OP NPs. The SAED image revealed four diffraction rings of CuO which were attributed to the (120), (002), (111) and (202) crystalline planes (Fig. 3d). However, the SAED image of CuO-OP showed various diffraction rings ascribed to (110), (002), (111), (202), (020), (202), (113), (311) and (200) which agreed with the XRD diffraction peaks (Fig. 4d).

### Batch adsorption experiments

#### pH effect

**Fig. 5:**
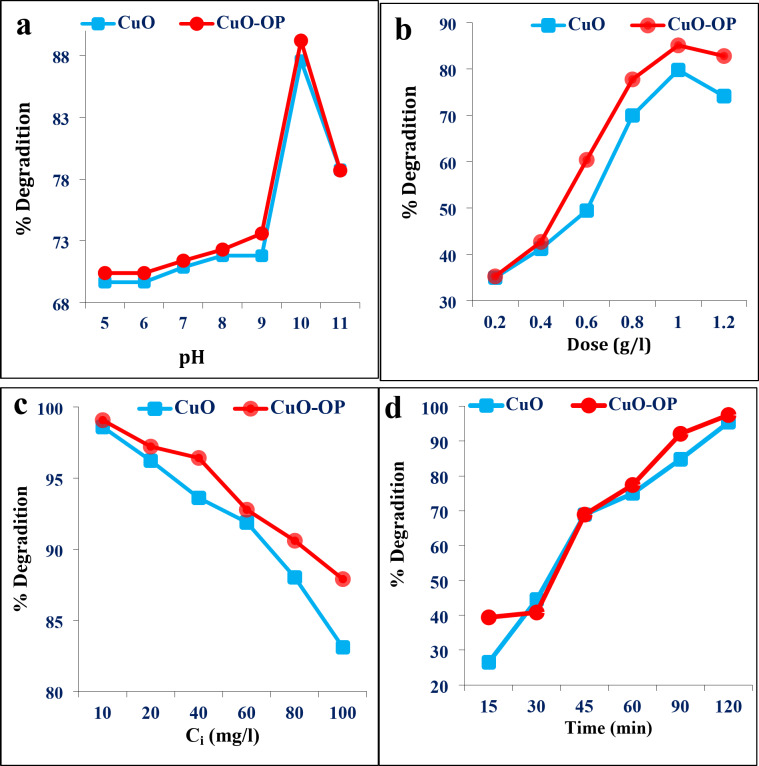
Effect of (**a**) pH, (**b**) catalyst dose (g/l), (**c**) initial dye concentration and (**d**) time (min) on the photodegradation of MB using CuO and CuO-OP NPs.

pH is the most important variable in adsorption and photocatalysis studies. pH strongly influences the catalyst surface load and ionization degree of the dye functional groups. Different pH values (6–11) were investigated to explore the photodegradation efficiency of MB using both CuO and CuO-OP NPs. Fig. 5a shows that increasing the pH from 6 to 10 enhanced the photodegradation percentage of MB from 69.66 to 87.58%, and from 70.4 to 89.2% for CuO and CuO-OP NPs, respectively. The maximum adsorption capacities (q_e_) were 87.58 and 89.20 mg/g for CuO and CuO-OP, respectively. Increasing the pH leads to an increase in the number of negative OH^−^ groups and more OH˙ radicals are available. These negative adsorbent sites cause electrostatic attraction with dye molecules; thus, the photodegradation efficiency increases. In comparison, Bassim et al.^47^ utilized an extract of *Citrus aurantium* to fabricate CuO/TiO_2_ and reported good photocatalytic degradation of 98.6% MB (11 mg/l) within 90 min (Table 2). Other studies reported optimum pH values for UV MB photodegradation of 10, 9, and 12 by Ali et al.^1,10^ and Kalaycıoğlu et al.^48^, respectively.

#### Catalyst dose

The dependence of MB photodegradation on the catalyst amount (0.2–1.2 g/l) was investigated. Increasing the catalyst dosage from 0.2 to 1 g/l significantly enhanced the photocatalytic degradation of MB from 34.99 to 79.78% for CuO NPs and from 35.26 to 85.1% for CuO-OP NPs. The addition of OP extract improved the photodegradation of MB dye (Fig. 5b). Nevertheless, increasing the catalyst dose beyond 1 g/L to 1.2 g/L led to a decrease in the photodegradation efficiency, this is likely attributed to the deactivation of excited catalyst molecules through collision with ground-state catalysts, consequently reducing the photodegradation process^49^. Nebal et al.^50^ referred to 30 mg/L as the optimum catalyst dose for 98% UV photodegradation of MB (5 ppm) by the green synthesized 2% CuO-ZnO (Table 2).

#### Initial dye concentration effect

Both CuO and CuO-OP NPs achieved maximum photocatalytic degradation rates of 98.6 and 99.08%, respectively of MB dye at the initial concentration of 10 mg/l. A remarkable decrease in MB photodegradation was noticed with gradual increasing of dye concentration. At a MB concentration of 100 mg/l (Fig. 5c), the % photodegradation decreased to 83.1 and 87.9% of CuO and CuO-OP, respectively. This may be due to lower light penetration at high dye concentrations in addition to fewer available active sites on the catalyst surface. Ali et al.^1^ reported that the photodegradation rate of MB gradually decreased from 98% at 5 mg/L to approximately 88% at 100 mg/L MB dye using CeO_2_ NPs. Murugan et al.^51^ and El-Katori et al.^52^ referred to 10 and 50 mg/L as the optimum concentration of MB dye using CeO_2_ and CdS/SnO_2_ NPs, respectively. Furthermore, Bassim et al.^53^ synthesized Fe_3_O_4_ using *Citrus aurantium* for the photocatalytic degradation of 93.14% MB through 43.71 min at an optimum dye concentration of 10 mg/l (Table 2).

#### Time effect

The Irradiation time is an important variable that governs photocatalytic degradation. Increasing the irradiation time from 5 min to 120 min, increases the dye photodegradation to 95.37 and 97.5% by CuO and CuO-OP NPs, respectively (Fig. 5d). Comparatively, Luque et al.^54^ synthesized SnO_2_ using orange peel extract (*Citrus sinensis*) and reported that the photocatalytic degradation of MB was 92% at an equilibrium time of 120 min (Table 2). Alkayakh et al.^49^ reported 240 min as the equilibrium time for MB photodegradation by MnTiO_3_/TiO_2_ and pure MnTiO_3_NPs. Ali et al.^1^ reported 90 min as the equilibrium time for the photodegradation of MB by pure CeO_2_ and CeO_2_@*Spirulena* NPs.

**Table 2:** Comparative study with other photocatalysts.

Catalyst	Dye	Initial dye concentration	Catalyst dose (mg)	pH	Time (min)	Degradation (%)	References
CuO	MB	100 mg/l	1000 mg/l	10	120	97.5	Present study
CuO-OP	MB	100 mg/l	1000 mg/l	10	120	95.37	Present study
CuO	RR195	50 mL of dye (10 mg/mL)	50 µg/mL	–	75	84.66	^37^
	RB			–		90.82	
**CuO**	CR	10 ppm	1 mg/ml	–	60	90	^55^
	NB	1 ppm	40 ppm	–	120	93	
**CuO**	MB	10 mg/l	20 ppm	–	135	92	^56^
	MR			–		85	
**CuO**	MB	1.0 × 10^− 5^ M	20 mg	–	120	87.37	^57^
				–		79.11	
				–		96.28	
**CuO**	MB	5 ppm	30 mg	–	90	52	^50^
**ZnO**						68	
**2% CuO-ZnO**						98	
**CuO nano bundles**	MG	–	–	–	70	98.20	^11^
**CuO/TiO**_**2**_	MB	10.93 mg/L	986.43 mg/l	8.87	90	98.6	^47^
**Fe**_**3**_ **O**_**4**_		10.02 mg/L	997.99 mg/L	8.98	43.71	93.14	^53^
**ZnO**	MB	15 mg/l	50	–	60	100	^54^
**SnO2**	MB	–	–	–	120	92	^58^
**ZnO**	MB	–	–	–	200	55	^59^
**Ag/ZnO**	MB	–	–	–	210	85	^60^
**ZnO**	MB	5	10 mg	10	150	92	^61^

– not available, *MB* Methylene blue,* RR195* Reactive RED 195,* CR* Congo Red,* MR* Methyl Red,* NB* Nile Blue,* MG* Malachite green,* RB* Rhodamine B.

### Isotherms studies

**Fig. 6:**
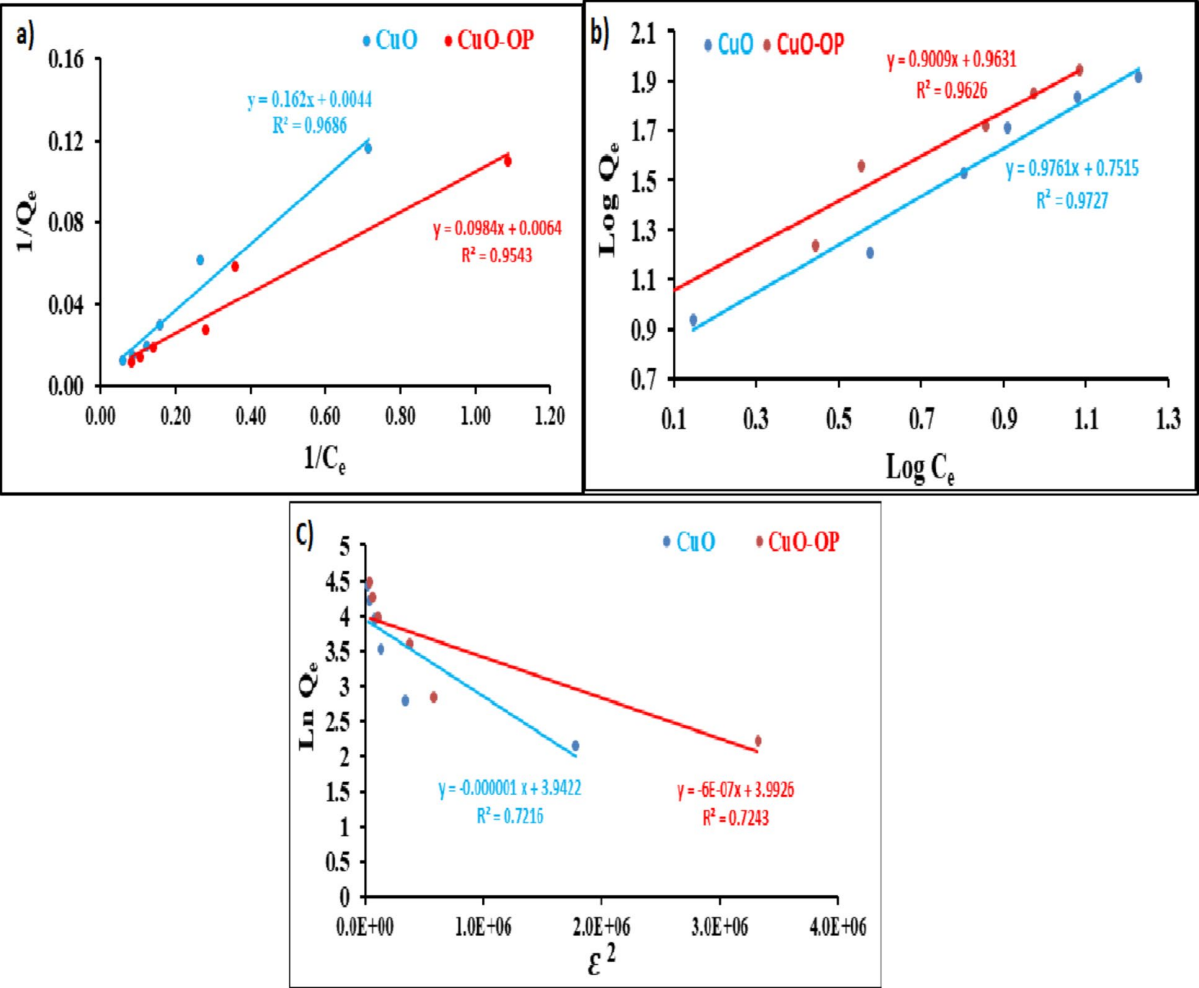
(**a**) Langmuir, (**b**) Freundlich and (**c**) D–R isotherms of MB photodegradation using CuO- and CuO-OP.

The Langmuir isotherm is a commonly used model that explains the formation of a monolayer of dye molecules on a solid surface^62^. Furthermore, the adsorption process proceeds on a homogeneous solid surface with similar sites and a similar level of energy^63^. Fig. 6a presents a plot of 1/Q_e_ vs. 1/C_e_ of the Langmuir model of MB photodegradation. A high correlation coefficient R^2^ of 0.97 and 0.96 was obtained when CuO and CuO-OP NPs were used, respectively. The values of the separation factor (R_L_) ranged from 0.268 to 0.134 (0 < R_L_ < 1), suggesting favorable photodegradation. The Langmuir maximum sorption capacities were 225.73 and 157.23 mg/g for CuO and CuO-OP NPs, respectively. Despite the high R^2^, the sorption capacities calculated via the Langmuir model differ somewhat from the experimental capacities.

The Freundlich model, which applies to the sorption mechanism within pores, describes the interactions between dye molecules and sorbents, assuming that there is a rapid decrease in the adsorption energy as the surface adsorption centers become depleted^64^. A plot of Log Q_e_ against Log C_e_ yielded higher R^2^ values of 0.97 and 0.96 for CuO, and CuO-OP NPs, respectively (Fig. 6b). The calculated Freundlich maximum sorption capacities (K_F_s) were 5.6 and 10.18 mg g^−1^ (L g^−1^)^1/n^. A higher K_F_ value of CuO-OP indicates greater sorption affinity and a higher photodegradation rate. The parameter “n” which describes the heterogeneity of the photocatalyst surface, had values of 1.02 and 1.11 for the CuO and CuO-OP NPs, respectively (Table 3). These values suggest a physical sorption process involving weak intermolecular forces^10^. Conversely, n values less than 0.5 indicate a low adsorption intensity^65^.

The D–R isotherm model was developed to consider the impact of the sorbent’s porous structure. It has been that the sorption process fills micropore areas, rather than proceeding layer by layer on pore walls^45^. This model outperforms the Langmuir model because it does not assume a uniform surface or consistent adsorption potential^66^. The maximum sorption capacities of the D–R model were calculated to be 51.52 and 54.22 mg g^−1^ for CuO, and CuO‒OP NPs, respectively. Similarly, the E values for photodegradation were estimated to be 707.1 and 912.9 KJ mol^−1^. E values less than 8 KJ mol^−1^ indicate physisorption. E values greater than 8 KJ mol^−1^ suggest chemisorption^67^. Therefore, the findings suggest that the photodegradation of MB onto CuO and CuO-OP NPs under UV irradiation involved chemisorption, with high energy values and good R^2^ values (0.71–0.73) (Fig. 6c).

**Table 3:** Isotherm variables for MB photodegradation by CuO, and CuO-OP.

Isotherm type	Variable	CuO	CuO-OP
Langmuir	Q_max_	225.73	157.23
	K_L_	0.027	0.065
	R_L_ (L mg ^−1^)	0.268	0.134
	R^2^	0.96	0.95
Freundlich	N	1.024	1.110
	K_f_ mg g ^−1^ (Lg ^−1^) ^1/n^	5.6	10.18
	R^2^	0.97	0.96
D–R	Q_max_	51.52	54.22
	E (KJ mol^−1^)	707.11	912.87
	K_ad_	1 × 10^− 6^	6 × 10^− 7^
	R^2^	0.72	0.72

### Kinetics studies

**Fig. 7:**
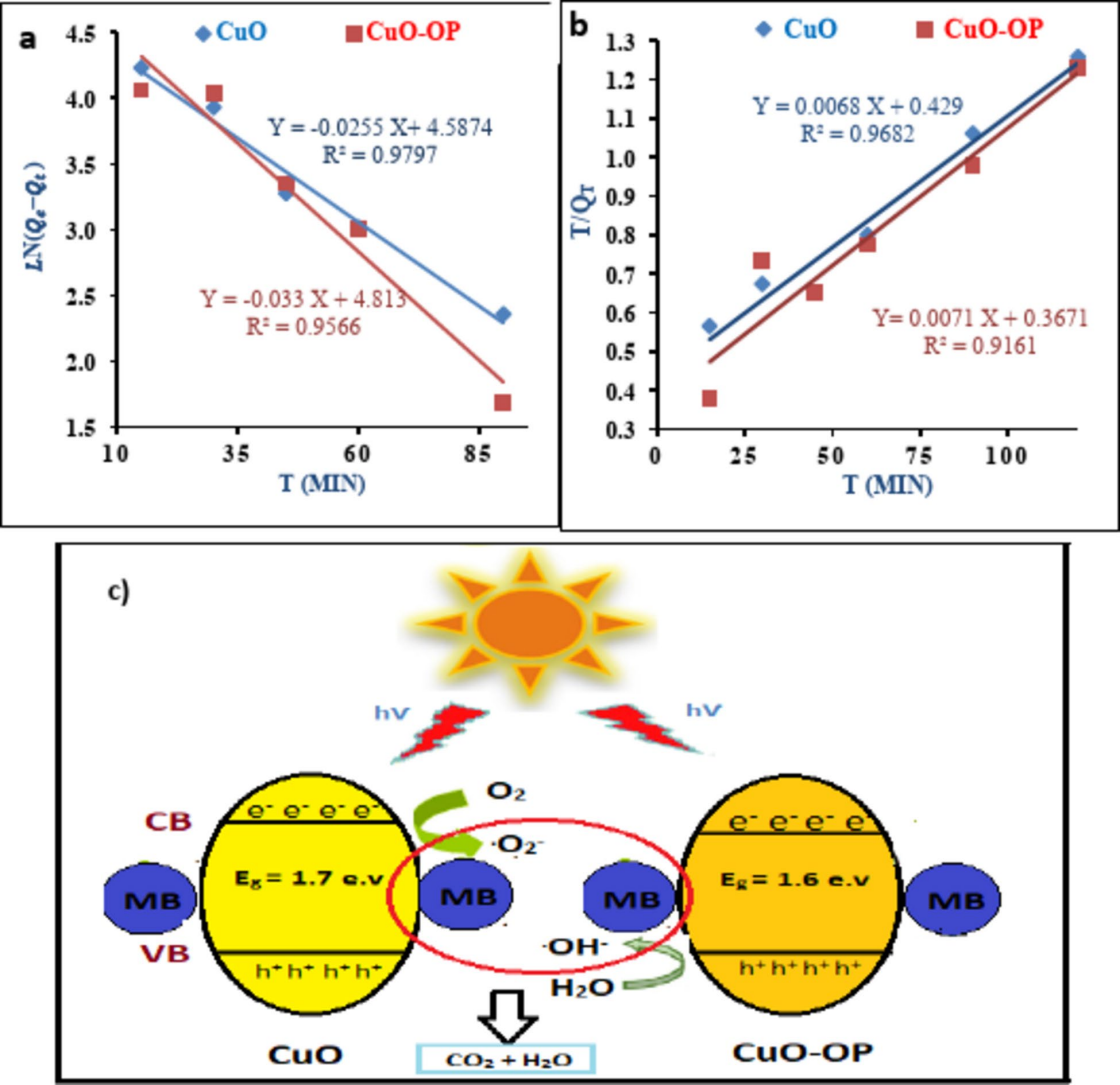
(**a**) Pseudo-first-order model, (**b**) pseudo-second-order model kinetics of the photodegradation of MB using CuO and CuO-OP NPs and (**c**) mechanism of photocatalytic degradation of MB.

The kinetic models, pseudo-first-order (PFO) and pseudo-second-order (PSO) are employed to elucidate the mechanisms, optimum conditions, and rate-controlling steps of MB degradation under UV irradiation using CuO and CuO-OP NPs. Table 4 presents the rate constants calculated from the experimental results. The calculated sorption capacities from PFO are 98.19 and 123.1 mg/g, with high R^2^ values of 0.98 and 0.96 for CuO and CuO-OP NPs, respectively. The rate constants (K_1_) were 0.0255 and 0.033 min^−1^ for CuO and CuO-OP NPs, respectively. The calculated q_max_ values from the PSO model were 147.56 and 141.24 mg g^−1^, with R^2^ values of 0.97 and 0.92 for CuO and CuO-OP NPs, respectively. The calculated q_max_ from PFO closely matched the experimental q_max_ values (95.37 and 97.5 mg/g) for both CuO and CuO-OP NPs. In addition, a higher R^2^ was computed from PFO than from PSO (Fig. 7a,b). On the other hand, a lower %SSE was computed from PSO than from PFO for both CuO and CuO-OP NPs (Table 4). After examining the experimental data using the PFO and PSO models, the PFO clearly and accurately characterized the photodegradation of MB onto the metallic oxides. This emphasizes the exceptional efficiency of the PFO model. CuO-OP results in a higher K_1_ value and better photocatalytic activity of the MB dye. Bassim et al.^47^ reported the photocatalytic decomposition of MB dye using CuO/TiO_2_ nanoparticles following the PFO kinetic model. In comparison, Nepal et al. studied the photodegradation kinetics of MB onto CuO@ZnO and reported PFO rate constants of 0.0093, 0.01419, 0.01572, 0.01919, 0.02014, and 0.04124 min^−1^ for CuO, ZnO, 50% CuO@ZnO, 25% CuO@ZnO, 5% CuO@ZnO, and 2% CuO@ZnO NCs, respectively^41^.

**Table 4:** Kinetics constant values of MB photodegradation using CuO and CuO-OP NP.

Order of reaction	Parameters	CuO	CuO-OP
	Q_e_ (mg/g)	95.37	97.5
PFO	Q_cal_ (mg/g)	98.19	123.1
	K_1_ (min ^−1^)	0.0255	0.033
	R^2^	0.98	0.96
	%SSE	0.05	0.16
PSO	Q_cal_ (mg/g)	147.56	141.24
	K_2_ (g mg^−1^ min^−1^)	0.00011	0.00014
	R^2^	96.82	91.61
	%SSE	0.01	0.04

Fig. 7c shows the proposed degradation mechanism of MB dye catalyzed by CuO and CuO-OP. Initially, the dye molecules are adsorbed onto the surface of the nanoparticle. Compared with CuO alone, the OP extract introduces additional functional groups to CuO-OP. These functional groups enhance the interactions with dye molecules through hydrophobic interactions and van der Waals forces, as recently reported^68^. Upon exposure to UV light with energy equal to or greater than the band gap energy (E_g_), the valence bond electrons in both CuO and CuO-OP are excited to the conduction band, forming electron ^_^ hole pairs. These electron ^_^ hole pairs undergo redox reactions with water molecules, producing hydroxyl radicals (^.^OH^−^) and superoxide (^.^O_2_^−^, Fig. 7c) under ambient conditions. These reactive species oxidize the chromophore groups in the dye molecule, breaking down the complex bonds responsible for its color, ultimately leading to decolorization of the dye solution^14^.

## Materials and methods

### Materials

High-purity anhydrous copper(II) acetate (CuC_4_ H_6_ O_4_, 99% purity), sodium hydroxide (NaOH, 99% purity), methylene blue dye (C_16_H_18_N_3_SCl, 98% purity), and absolute ethanol (C_2_H_6_O) were acquired from Sigma Aldrich. Deionized water was used to prepare both the extract and the methylene blue (MB) solutions. All the reagents were analytically pure and were not further purified before use. The deionized water used in this work was ultrapure.

### Extraction of the orange peel

Orange fruits (*Citrus reticulata*) were purchased from a local market in Egypt. The Orange Peel (OP) was pulverized into small pieces and thoroughly washed with hot distilled water several times. The OP was dried at 45 ⁰C for four days to avoid volatilization of the active ingredients, ensuring its efficacy and stability. The dry OP was ground in an electric mill and sieved through a sieve of 63 μm. Approximately 40 g of OP powder was added to a 400 ml mixture of ethanol and water (1:3 V/V). The mixture was heated at 70 ⁰C with magnetically stirred at 600 rpm. After that, it was centrifuged at 10,000 rpm for 15 min to obtain OP^20^. The extract is stored at − 4 °C for further use.

### CuO synthesis

**Fig. 8:**
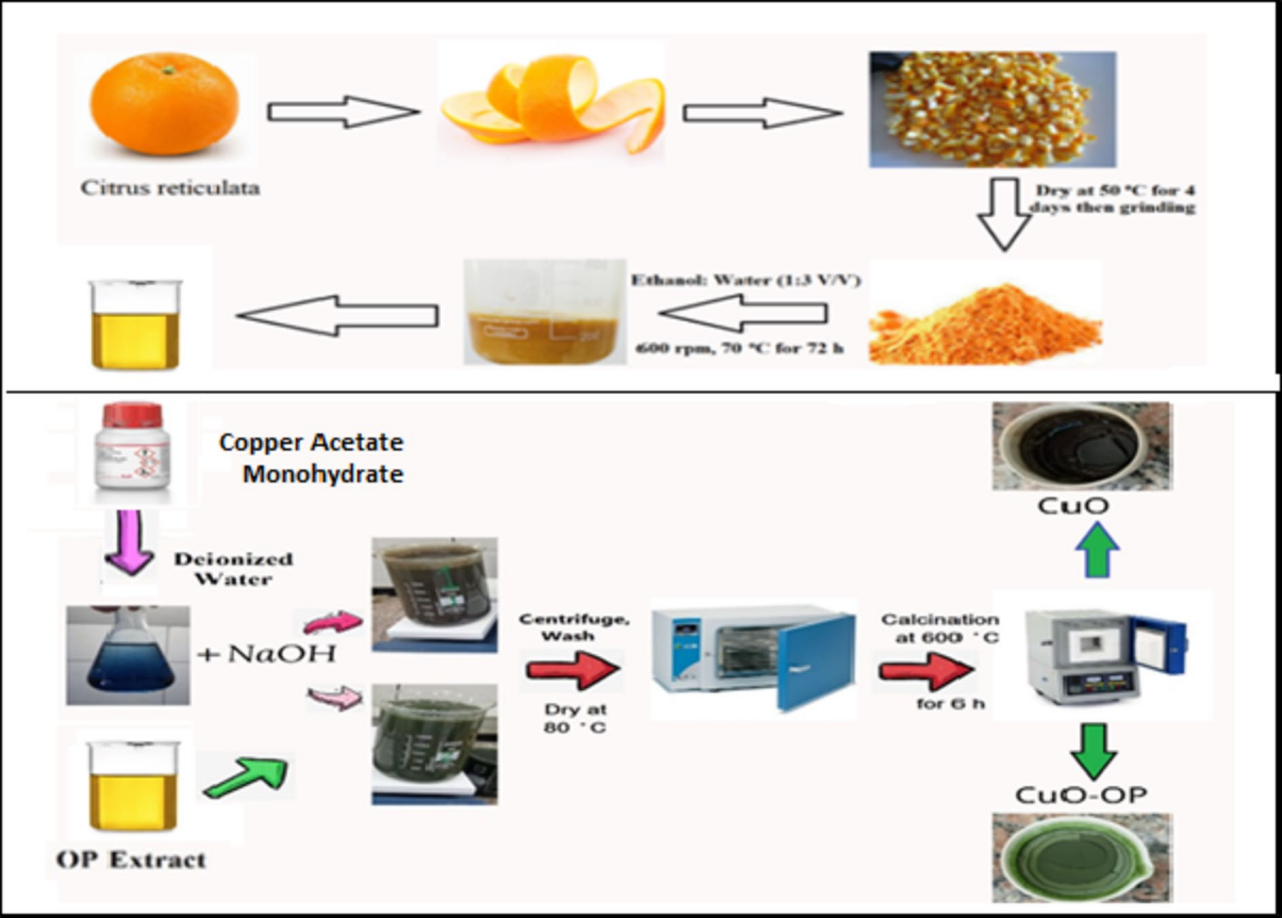
Schematic diagram illustrates the preparation of CuO, and CuO-OP NPs.

Initially, 33.4 g of copper acetate monohydrate (0.8 M) was completely dissolved in deionized water with stirring at 80 °C for 1 h. Next, NaOH solution (0.8 M) was added dropwise to the solution with stirring at 80 °C until a complete Cu(OH)_2_ precipitate formed. The resulting mixture was stirred for an additional 24 h. To prepare the green CuO NPs, the OP extract was slowly added to the Cu^2+^ solution with a solution of sodium hydroxide with stirring at 80 °C (Fig. 8). The resulting mixture was magnetically stirred for 24 h. Subsequently, a rapid centrifugation at 8,000 rpm for 15 min to separate the precipitate. The precipitate was dried in an oven at 100 °C and calcined at 600 °C for 6 h using a muffle furnace (model: Nabrotherm p 180, Germany).

### Physical characterizations

#### The N_2_ adsorption/desorption measurements

The surface area was measured using the Brunauer–Emmett–Teller (BET) method by analysing the nitrogen adsorption isotherm. The distribution of pore size was measured through the desorption branch of Barrett–Joyner–Halenda (BJH) isotherm analysis.

#### Ultraviolet-visible diffuse reflectance spectroscopy

Spectroscopy of UV–VIS diffuse reflectance (DRS) was performed using a Perkin–Elmer Lambda-900 spectrophotometer at wavelengths ranging from 200 to 800 nm. The UV‒VIS spectra were analysed via Microsoft Excel software to perform data processing.

#### X-ray diffraction

The grain size and crystal structure of the produced oxides were determined using X-ray diffraction (XRD). XRD measurements were conducted using Philips, PW-371 diffractometer, employing Cu-Kα radiation (λ = 1.54056 Å). The samples were scanned across a 2θ angle range of 20 to 80 degrees at a rate of 5 degrees per minute, using a tube voltage of 40 kV and a current of 30 mA. The average crystalline sizes of the CuO and CuO-OP NPs were calculated via the Debeye–Scherer equation (Eq. 3).

3 $$D= \frac{K\lambda }{\beta Cos\theta }$$

where D is the average crystallite size, K is the shape factor (0.94), λ is the X-ray wavelength (Cu Kα = 1.5406 Å), θ is the Bragg angle, and β is the full width at half maximum (FWHM/2).

#### Fourier transform infrared (FTIR) spectra

Fourier transform infrared (FTIR) spectroscopy analysis was conducted to identify the functional groups within the wavenumber ranging between 4000 and 400 cm^−1^. The samples were mixed with KBr, pressed into a pellet and analysed via a Perkin-Elmer device (Spectrum GX).

#### Scanning electron microscopy and energy dispersive X-ray spectroscopy

The physical structure and elemental composition of the as-synthesized catalyst were examined using scanning electron microscopy (SEM) coupled with energy-dispersive X-ray spectroscopy (EDX) using a JEOL JEM 2100 F UHR microscope operating at 200 KeV.

#### Transmission electron microscopy

The morphology and particle size of the as-synthesized catalyst were characterized using High-resolution transmission electron microscopy (HRTEM) using the Tecnai G2 Supper Twin instrument.

### Photocatalytic degradation experiments

The photocatalytic degradation experiments were performed under a UV light source comprised of 6 mercury lamps, each having a power output of 11 watts and emitting light at a wavelength of 260 nm, with a light intensity of 50 lx. A stock solution of methylene blue dye (MB) with a concentration of 1000 mg/L was utilized to prepare a standard working solution with a concentration of 100 mg/L. Fifty milligrams of photocatalyst were dispersed in a 50 mL solution of MB (100 mg/L). The mixture was stirred in the dark for 30 min until it reached adsorption-desorption equilibrium. The effects of pH, catalyst dosage, irradiation time, and initial MB concentration were examined. The photocatalyst samples were separated by centrifugation, and the remaining MB concentration in the resulting supernatants was determined using a Jenway 6800 UV-VIS spectrophotometer. The photocatalytic capacity (Q_e_ mg/g) and removal efficiency (R) were calculated using Eqs. (4) and (5).

4 $${{Q}}_{{e}}= \frac{{({C}}_{0 }- {{C}}_{{e}}) \times {V}}{{M}}$$

5 $${\%} {R}= \frac{{({C}}_{0 }- {{C}}_{{e}}) \times 100}{{{C}}_{0}}$$

Where q_e_: photocatalytic capacity (mg/g), R: removal efficiency (%), V: volume of MB dye (L); C_0;_ initial MB concentration; C_e_; the equilibrium MB concentration, and M; the catalyst mass (g).

### Isotherm models

Adsorption isotherms are essential for developing effective adsorption systems for environmental purposes. They provide information regarding the adsorption mechanisms, either monolayer or multilayer adsorption, and they allow for predicting the maximum adsorption capacity. Langmuir, Freundlich, and Dubinin–Radushkevich isotherm models have been investigated. The mathematical formulae employed in calculating the adsorption capacity and the isotherm constants are presented in Table 5.

### Isotherm kinetics

Studying the adsorption kinetics allows for the determination of both the adsorption rate and the rate-limiting stages. The rate-limiting step can contribute to improving the catalyst efficiency. The adsorption process has been modelled using the pseudo-first-order (PFO) and pseudo-second-order (PSO) kinetic models (Table 5).

**Table 5:** The equations and symbol definitions employed in the Isotherm models.

Model	Equation	Symbols definition
Isotherm models		
Langmuir	$\frac{{C}_{e}}{{Q}_{e}}= \frac{1}{{K}_{L}\times {Q}_{m}}+ \frac{{C}_{e}}{{Q}_{m}}$ ${R}_{L}= \frac{1}{1+{K}_{L}{C}_{i} }$	Q_m_: the adsorption capacity in mg/g, C_e_: the equilibrium concentration in mg/L, K_L_: Langmuir constant (L/mg), R_L_ is the separation factor, R_L_ > 1, unfavourable; R_L_ = 1, linear; 0 < R_L_ < 1, favourable; R_L_ = 0, irreversible adsorption.
Freundlich	${Q}_{e}={K}_{F}\times {C}_{e}^{1/n}\text{log}{Q}_{e}=\text{log}K+ \frac{1}{n} \times \text{log}{C}_{e}$	K_F_ and n are Freundlich constants.
Dubinin–Radushkevich	$\text{ln}{Q}_{e }= \text{ln}{Q}_{m}-{K}_{ad }{\varepsilon }^{2}$ $\varepsilon =RT\text{ln}(\frac{1}{1+ {C}_{e}})$ $E=\frac{1}{\sqrt{2{K}_{ad}}}$	$\varepsilon$: is the Polanyi potential, ${K}_{ad }$ (mol^2^ kJ^− 2^) is a constant related to the adsorption energy (E). E values < 8 refer to physisorption, 16 > E > 8 refers to chemisorption reaction.
Kinetic models		
Pseudo first order	$Log\left({Q}_{e}-{Q}_{t }\right)=\text{log}{Q}_{e}-\frac{{K}_{1 \times }t}{2.303}$ $\frac{1}{{Q}_{t}}=\frac{{K}_{1}}{{Q}_{e }\times t}+\frac{1}{{Q}_{e}}$	K_1_ the first order rate constant (min^-1^).
Pseudo second order	$\frac{t}{{Q}_{t}}= \frac{1}{{K}_{2}\times {Q}_{e}^{2}}+\frac{t}{{Q}_{e}}$	K_2_ the second order rate constant (g mg^-1^ min^-1^).

## Conclusion

This study utilized an eco-friendly green synthesis approach using *Citrus aurantium* extract to prepare CuO nanoparticles. The textural, optical and nanostructural properties of the CuO and CuO-OP were characterized using DRS, HRTEM, N_2_-adsorption desorption isotherms, SEM-EDX, XRD, and HRTEM. The XRD, SEM, and HRTEM results revealed that the nanoparticles exhibited monoclinic cubic and spherical morphologies. FTIR confirmed the presence of Cu-O bonds in the samples. The specific surface area (S_BET_) indicated a homogeneous pore size distribution ranging from mesopores to macropores upon the addition of the OP extract. The DRS results depicted band gap energies of 1.7 and 1.6 eV for CuO and CuO-OP, respectively, as determined by the Tauc plot. The selected area electron diffraction (SEAD) pattern exhibited circular rings, confirming the polycrystalline nature of both the CuO and CuO-OP NPs. SEM images showed that the addition of OP extract to CuONPs introduced surface cavities on the CuO-OP surface that improved the photocatalytic activity. EDX measurements revealed the incorporation of C (1.6%) and N (0.3%) from the orange peel extract into the green CuO-OP NPs. Both chemically and green CuO NPs exhibited excellent degradation efficiency of methylene blue dye under UV light, reaching 95.43 and 97.5%, respectively. The optimal conditions for the degradation process included an initial dye concentration of 100 mg/L, a pH of 10, a catalyst dosage of 1 g/L, and a contact time of 120 min. The adsorption process was better described by the Freundlich isotherm model (R^2^ = 0.97 and 0.96) than the Langmuir model. The energy values for photodegradation calculated from D–R isotherm were 707.11 and 912.87 KJ mol ^−1^, referred to as chemisorption. The calculated sorption capacities from PFO were 98.19 and 123.1 mg/g with high R^2^ values of 0.98 and 0.96 for CuO and CuO-OP NPs, respectively. The rate constants (K_1_) were 0.0255 and 0.033 min^−1^ for CuO and CuO-OP NPs, respectively.

## Data Availability

Data is provided within the manuscript.
